# Transient Anti-Glutamic Acid Decarboxylase (GAD) Antibody Positivity and Insulin Secretion Reduction After IVIg in Type 2 Diabetes Mellitus

**DOI:** 10.7759/cureus.82038

**Published:** 2025-04-10

**Authors:** Hirotaka Nakashima, Takuya Omura, Xiaofu Teng, Takahiro Kamihara, Katsunori Yokoi, Shuji Kawashima, Haruhiko Tokuda

**Affiliations:** 1 Department of Endocrinology and Metabolism, National Center for Geriatrics and Gerontology, Obu, JPN; 2 Department of Internal Medicine, Minami Seikyo Hospital, Nagoya, JPN; 3 Department of Metabolic Research, Research Institute, National Center for Geriatrics and Gerontology, Obu, JPN; 4 Department of Internal Medicine, National Center for Geriatrics and Gerontology, Nagoya, JPN; 5 Department of Cardiology, National Center for Geriatrics and Gerontology, Obu, JPN; 6 Department of Neurology, National Center for Geriatrics and Gerontology, Obu, JPN; 7 Department of Clinical Laboratory, National Center for Geriatrics and Gerontology, Obu, JPN

**Keywords:** autoantibodies, glutamate decarboxylase, hyperglycemia, intravenous immunoglobulins, type 1 diabetes mellitus, type 2 diabetes mellitus

## Abstract

This report describes a patient with type 2 diabetes mellitus who received intravenous immunoglobulin therapy for Guillain-Barré syndrome and subsequently experienced a sudden deterioration in glycemic control. Although anti-glutamate decarboxylase antibodies became positive and insulin secretion was transiently suppressed, both findings returned to baseline during the follow-up period. These changes initially raised concerns about new-onset or coexisting type 1 diabetes mellitus; however, the reversibility of anti-glutamate decarboxylase antibody positivity and the eventual restoration of insulin secretion argued against a new or evolving form of autoimmune diabetes. In type 2 diabetes, chronic inflammation may predispose individuals to immune dysregulation, and receiving intravenous immunoglobulin could transiently exacerbate underlying metabolic instability. In this case, however, the marked hyperglycemia and the brief period of suppressed insulin secretion did not lead to sustained autoimmune activity or permanent β-cell dysfunction. Instead, the patient’s anti-glutamate decarboxylase levels reverted to normal, insulin production capacity improved, and classification as type 2 diabetes mellitus was ultimately reaffirmed. These findings underscore the need for clinicians to remain vigilant when interpreting new-onset antibody positivity or metabolic disturbances that arise shortly after intravenous immunoglobulin therapy. Repeated assessment of both antibody levels and insulin secretion is crucial to avoid premature changes in diabetes classification or therapeutic strategies based solely on transient antibody elevation. This case highlights the importance of recognizing that anti-glutamate decarboxylase antibody positivity and corresponding hyperglycemia can be induced transiently by immunoglobulin therapy and do not necessarily indicate progression to type 1 diabetes mellitus. Regular monitoring of metabolic parameters and clinical status over time can ensure more accurate diagnosis and management in patients with preexisting diabetes who experience sudden, unexplained changes in blood glucose following immunoglobulin administration.

## Introduction

Intravenous immunoglobulin (IVIg) is predominantly administered to treat autoimmune diseases. A previous study reported transient increases in the levels of various autoantibodies after IVIg administration [1]. The proposed mechanisms of these transient autoantibody elevations include passive transfer of antibodies from IVIg and temporary stimulation of antibody production. Herein, we report a patient with type 2 diabetes mellitus who experienced sudden deterioration in blood glucose control after receiving IVIg for Guillain-Barré syndrome (GBS). The clinical significance of IVIg-induced autoantibodies remains unclear; however, we encountered a case in which the patient developed metabolic abnormalities resembling those seen in type 1 diabetes mellitus.

## Case presentation

An 86-year-old woman diagnosed with type 2 diabetes mellitus received oral hypoglycemic treatment at a local clinic. The patient had a medical history of hypertension, dyslipidemia, lumbar spinal canal stenosis, and osteoporosis. Her diabetic complications included vitreous hemorrhage due to diabetic retinopathy and early-stage diabetic nephropathy; diabetic neuropathy was absent. She was independent in activities of daily living and ambulation with the help of a cane.

Admission

The patient experienced persistent lower limb muscle weakness, resulting in hospital admission after four days. On admission, she was alert, with a blood pressure of 143/98 mmHg, a heart rate of 63 bpm, a blood glucose level of 170 mg/dL, and a glycosylated hemoglobin (HbA1c) level of 7.3%. She was diagnosed with GBS and received IVIg therapy for five days.

**Figure 1:**
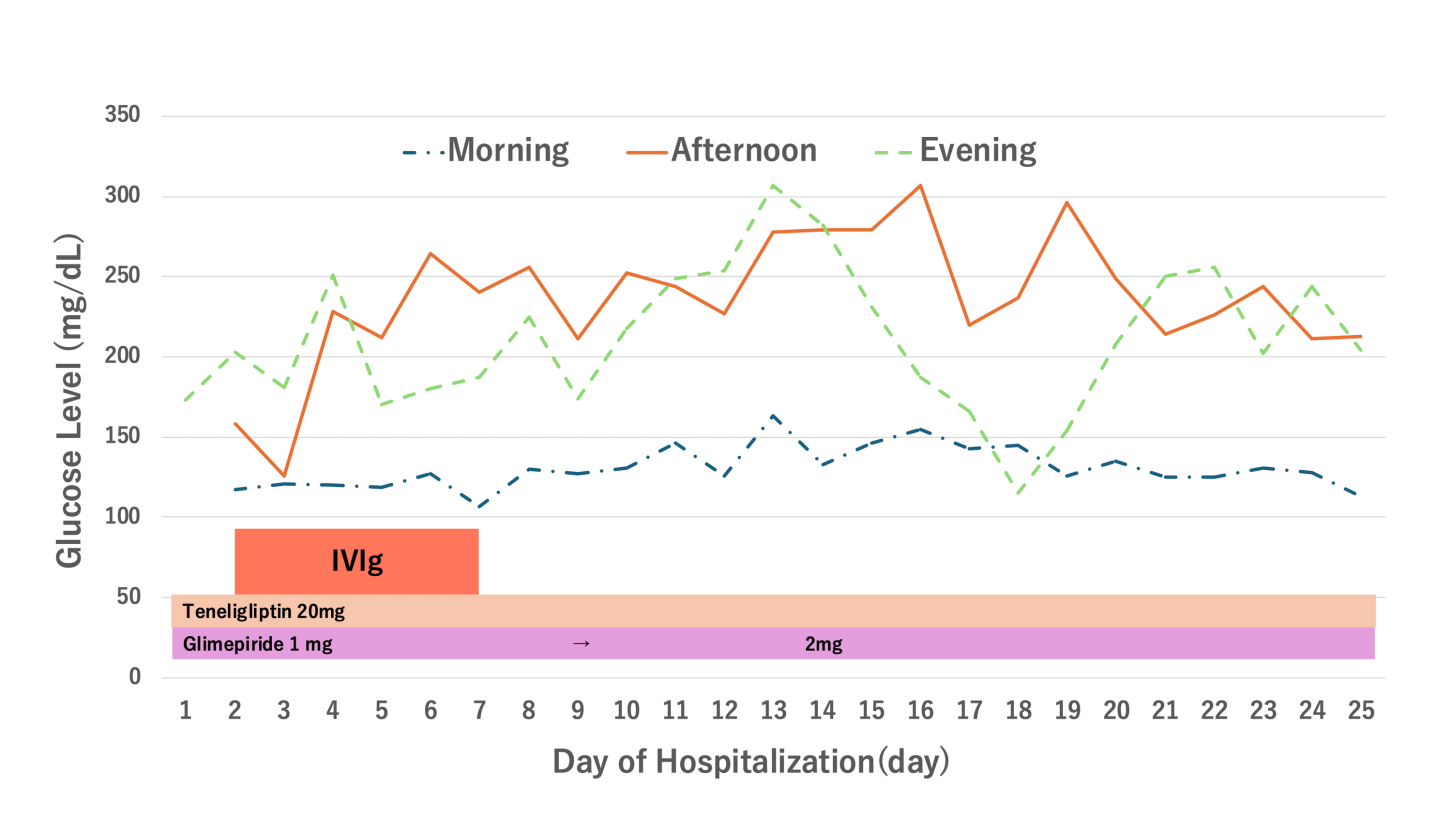
Trends in blood glucose levels over time Blood glucose levels remained stable before IVIg administration. However, worsening glycemic control was observed three days after IVIg therapy initiation, followed by fluctuations. The morning, afternoon, and evening glucose levels are presented separately. Treatment details, including IVIg and antidiabetic medications, are indicated below the graph.

Despite the pretreatment blood glucose levels remained generally within the normal range after admission, a rapid increase in blood glucose was observed three days after IVIg initiation (Figure 1).

Approximately two weeks after admission, urinary C-peptide levels were 33.98 µg/day, indicating reduced insulin secretion. Although anti-glutamic acid decarboxylase (GAD) antibody testing performed 11 years prior was negative, repeat testing on the current day yielded a positive result of 39.9 U/mL (Table 1).

**Table 1:** Longitudinal changes in insulin secretion and GAD antibody levels GAD antibody level was 39.9 U/mL, and the urinary C-peptide level was 33.98 µg/day 2 weeks after admission. The GAD antibody became undetectable within three months. The GAD antibody remained at <5.0 U/mL at 27 months, and the urinary C-peptide was 93.3 µg/day, indicating preserved β-cell function. †Serum insulin levels were measured incidentally in another department at approximately 2 and 14 months.

Months since Admission(month)	0.5	2	3	14	27	Reference ranges
GAD antibody (U/mL)	39.9	-	<5.0	-	<5.0	0.0-4.9
Urinary C-peptide (μg/day)	33.98	-	-	-	93.3	29.2-167
Serum insulin (μU/mL)†	-	12.7	-	31.9	-	2.7-10.4

Other potential factors that could contribute to decreased insulin secretion, such as pancreatic disease, malignancy, infection, or corticosteroid administration, were not identified. Based on these results, slowly progressive insulin-dependent diabetes mellitus (SPIDDM) was considered as a differential diagnosis instead of type 2 diabetes, thereby prompting adjustments to the patient’s oral hypoglycemic agents.

After completing the initial treatment for GBS, insulin therapy was planned for outpatient management, and the patient was discharged home.

Follow-up

**Figure 2:**
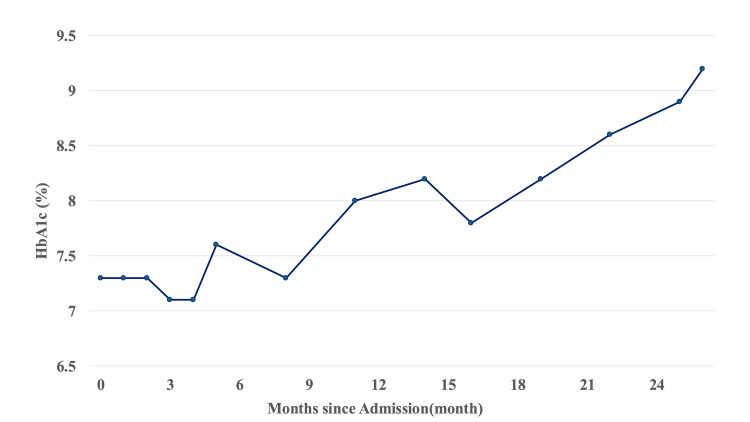
Time course of HbA1c levels HbA1c levels remained relatively stable for the first six months after admission, then gradually increased to 9.2% during outpatient follow-up.

The anti-GAD antibody was negative within three months, indicating that SPIDDM was unlikely. The patient was subsequently managed for type 2 diabetes. During the outpatient follow-up, HbA1c remained between 7% and 8% but eventually increased to 9.2% approximately two years after discharge (Figure 2).

However, the anti-GAD antibody remained negative, and the urinary C-peptide was 93.3 µg/day, indicating preserved insulin secretion (Table 1). These results further indicated that the patient did not have SPIDDM. Subsequently, the patient’s blood glucose level improved.

## Discussion

Previous studies have reported transient autoantibody positivity after IVIg administration. Various autoantibodies, including SS-A, Tg, and TPO, were detected [1].

Autoantibody screening performed immediately after IVIg administration revealed (1) passively transferred antibodies or (2) transiently induced antibody production that generated positive test results. This possibility requires careful interpretation, particularly when assessing connective tissue diseases and antiphospholipid antibody syndrome.

The first proposed mechanism indicates that autoantibodies from blood donors are transferred through IVIg, which consists of pooled plasma [1]. The second proposed mechanism indicates that IVIg stimulates B cells, thereby increasing plasmablasts and transient antibody production [2]. This is further supported by reports showing that post-IVIg autoantibody levels sometimes exceed the expected values based on the antibody concentrations in IVIg [1].

Moreover, a previous study reported the development of anti-GAD antibody positivity after IVIg administration [1]. Approximately 1%-2% of healthy individuals test positive for anti-GAD antibodies [3,4], which may also be present in IVIg [5].

GAD, expressed in pancreatic β-cells, plays a role in γ-aminobutyric acid (GABA) and regulation of insulin secretion through GABA receptor signaling [6,7]. Anti-GAD antibodies inhibit GAD activity, thereby impairing insulin secretion [8]. In contrast, anti-GAD antibodies present in IVIg are potentially nonpathogenic and naturally decline within approximately 3-4 weeks [1,5]. However, distinguishing between passive transfer of antibodies and transiently induced antibody production remains challenging. The relative contribution of each mechanism may vary depending on the patient’s underlying immune status and clinical background. Chronic inflammation associated with diabetes may have primed the immune system to transiently produce increased amounts of autoantibodies after IVIg administration. Although we did not measure the concentration of autoantibodies contained in the IVIg administered to this patient, previous studies have reported antibody titers in IVIg preparations [1]. When comparing the estimated total amount of antibodies administered via IVIg with the total amount of antibodies detected in the patient's circulation, the latter appears to be substantially greater, suggesting that passive transfer alone cannot fully explain the observed antibody levels. This strongly supports the hypothesis of active, transient antibody production following IVIg therapy.

This case presents a rare opportunity to investigate the dynamic changes in insulin secretion, including its transient suppression and subsequent recovery. In this patient, glycemic control was stable during type 2 diabetes mellitus treatment, but it rapidly deteriorated after IVIg administration. During this period, C-peptide levels were relatively suppressed despite worsening hyperglycemia. However, insulin secretion gradually increased in the later phase following worsening glycemia. Hence, the following hypothesis was proposed: This abrupt worsening of hyperglycemia coincided with the emergence of anti-GAD antibodies, indicating the possibility that the transient autoantibody response contributed to impaired insulin secretion.

Moreover, a notable feature was the spontaneous disappearance of anti-GAD antibodies within three months. In contrast, SPIDDM is characterized by persistently increased anti-GAD antibody levels over several years, progressive β-cell dysfunction, and eventual insulin dependence [9]. These results indicate that the diagnosis of type 2 diabetes mellitus was more appropriate than the diagnosis of type 1 diabetes mellitus in our patient. Therefore, the clinical progression and insulin secretion capacity should be carefully monitored rather than immediately diagnosing type 1 diabetes when anti-GAD antibodies become positive after IVIg therapy. To prevent misclassification of diabetes type and the resulting adverse effects on clinical management, autoantibody testing after IVIg therapy should be considered only when significant hyperglycemia is observed. If GAD antibodies are positive, we recommend retesting at least one month after IVIg therapy to determine whether the positivity is transient.

However, this is a single case; thus, the frequency, duration, and metabolic effects of transient autoantibody positivity after IVIg therapy remain poorly understood. To the best of our knowledge, no cases of new-onset diabetes in individuals without diabetes after IVIg therapy have been reported. Chronic inflammation is associated with metabolic stress and may play a role in immune dysregulation in type 2 diabetes mellitus [10,11]. These results indicate that IVIg-induced transient hyperglycemia and autoantibody positivity are more likely to manifest clinically in patients with diabetes mellitus due to their β-cell vulnerability. In contrast, in individuals without diabetes, differences in immune response thresholds may mitigate the risk of hyperglycemia or diabetes-related onset. Further studies are warranted to clarify the clinical significance of transient autoantibody positivity and its potential effect on glucose metabolism in patients receiving IVIg therapy.

## Conclusions

This case highlights the possibility of transient autoantibody positivity and hyperglycemia after IVIg therapy. However, autoantibody positivity does not necessarily indicate the progression to type 1 diabetes mellitus. In diabetes management and classification, clinicians need to assess not only fluctuations in antibody levels but also insulin secretion capacity and the overall clinical course.
